# Radiological complete response with regorafenib for multiple lung metastases of ascending colon cancer: a case report

**DOI:** 10.1186/s13256-023-04337-7

**Published:** 2024-02-07

**Authors:** Koji Kikuchi, Masaaki Ogawa, Akira Sasaki

**Affiliations:** 1 Department of Surgery, Iwate Medical University, 2-1-1 Idaidori, Yahaba, Iwate 028-3695 Japan; 2 Department of Surgery, Kazuno Kosei Hospital, 18 Mukaibatake, Hanawa, Kazuno, Akita 018-5201 Japan

**Keywords:** Regorafenib, Multiple lung metastasis, Colorectal cancer, Radiological complete response

## Abstract

**Background:**

Regorafenib is an oral diphenylurea multikinase inhibitor and currently approved for use following third-line therapy for metastatic colorectal cancer (CRC) patients. Only one case has previously been reported of metastatic CRC showing a complete response (CR) to regorafenib.

**Case presentation:**

A 68-year-old Japanese man underwent laparoscopy-assisted ileocecal resection and D3 lymphadenectomy due to his ascending colon cancer. Eighteen months after surgery, a laparoscopic hepatic left lateral segmentectomy was performed due to a liver tumor, and a pathological diagnosis of colorectal liver metastasis was made. Three months after the second surgery, contrast-enhanced computed tomography (CT) revealed multiple lung metastases. The patient had undergone 18 courses of the FOLFOX + bevacizumab chemotherapy regimen as their first-line therapy and 11 courses of the FOLFIRI + ramucirumab chemotherapy regimen as their second-line therapy. As their third-line therapy, the patient was administered the regorafenib chemotherapy regimen. We evaluated the chemotherapy treatment’s effect on the lung tumors by CT after 3, 7, 11, and 17 courses of the regorafenib chemotherapy regimen, finding that the lung tumors had shrunk with time; thus, each tumor was considered a partial response (PR) based on the RECIST guidelines. After 21 courses of the regorafenib chemotherapy regimen, the chemotherapy was discontinued in response to the patient’s wishes. Even at 1 and 3 months after the discontinuation of the chemotherapy, CT revealed that the lung tumors had shrunk, with each considered a PR. Furthermore, 9 months after the discontinuation of the chemotherapy, CT revealed scarring of the lung tumors. This was considered a CR, rather than a PR. The patient remains disease-free 18 months after the discontinuation of chemotherapy.

**Conclusions:**

In this paper, we present the second case of radiological CR with regorafenib for multiple lung metastases of ascending colon cancer. Currently, there is no consensus on a treatment strategy for patients with radiological CR.

## Background

Colorectal cancer (CRC) manifests as malignant tumors that are among the most commonest causes of cancer-related deaths. Metastasis is a primary contributor to CRC-related mortality, with the liver and lungs representing the most frequently involved organs. For metastatic or unresectable CRC, standard first- and second-line treatments typically involve a combination of cytotoxic chemotherapies and molecular targeted agents that can help to improve progression-free survival and overall survival [1–3].

Regorafenib is currently approved for use following third-line therapy for metastatic CRC patients [4]. Regorafenib is an oral diphenylurea multikinase inhibitor that targets angiogenic (vascular endothelial growth factor receptor 1–3 and TIE2), stromal (platelet-derived growth factor receptor and fibroblast growth factor receptor), and oncogenic receptor tyrosine kinases (KIT, RET, and RAF) [5]. The CORRECT and CONCUR trials indicated that regorafenib facilitates overall survival and progression-free survival (PFS) when compared to a placebo in metastatic CRC patients; however, disease control (partial response [PR] plus stable disease) was achieved in 51% of the patients, 4% showed a PR, and none showed a complete response (CR) [4, 6]. Hyungjoo et al. recently reported the first case of metastatic CRC that showed a CR to regorafenib [7], representing is the only report showing a CR in a metastatic CRC patient. In this report, we present a case of radiological CR with regorafenib for multiple lung metastases of ascending colon cancer.

## Case presentation

**Fig. 1:**
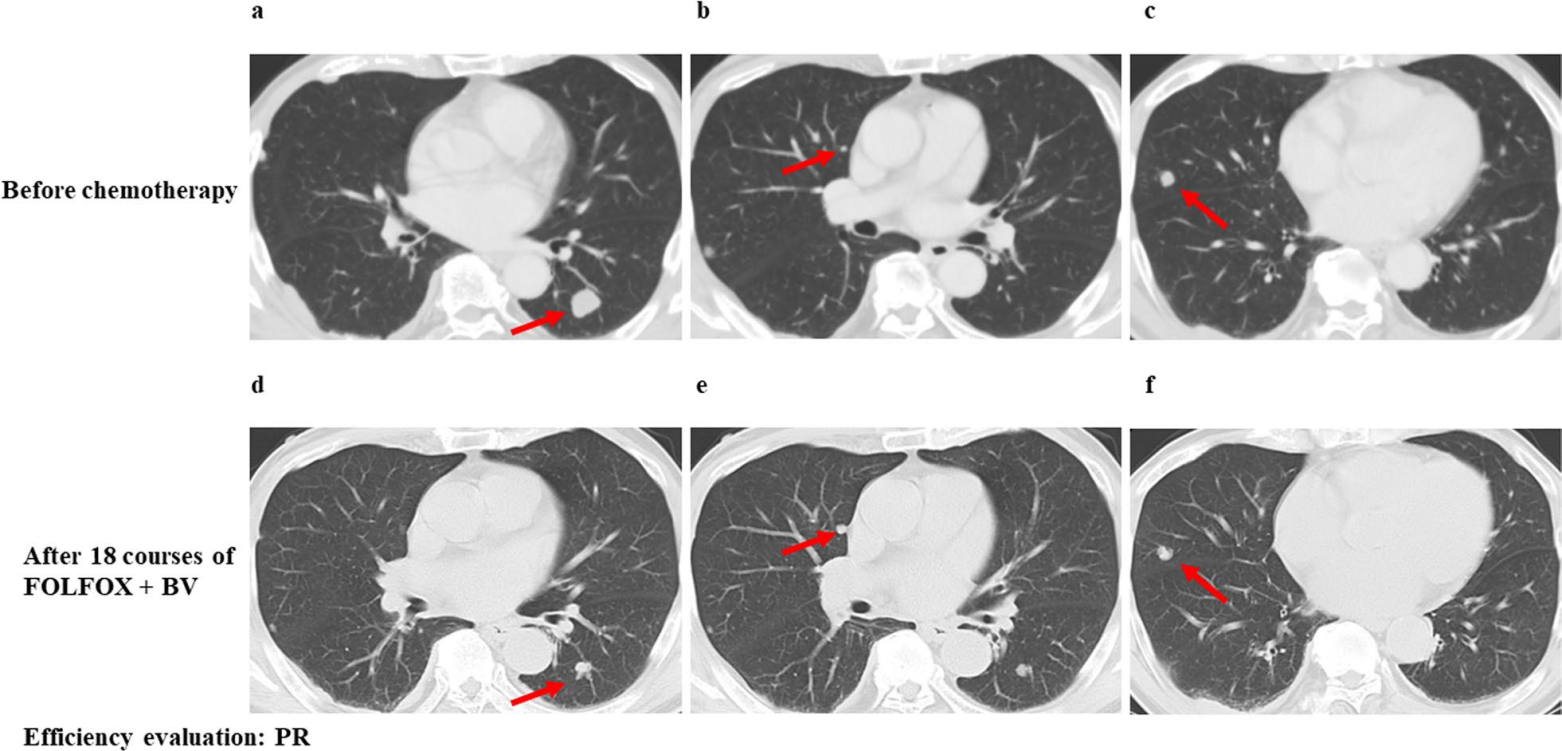
Consecutive CT findings before chemotherapy and after first-line chemotherapy. **a**–**c** Multiple lung metastases were revealed. **d**–**f** The lung tumors had shrunk after 18 cycles of the FOLFOX + BV chemotherapy regimen had been administered. These arrows indicate lung metastases

A 68-year-old Japanese man underwent laparoscopy-assisted ileocecal resection and D3 lymphadenectomy due to his ascending colon cancer in October 2016. The pathology revealed a stage IIIa moderately differentiated *KRAS* gene mutation in codon 146 and BRAF V600E wild-type adenocarcinoma, with two out of 17 lymph nodes testing positive. Microsatellite instability (MSI) testing revealed low-level MSI. The patient had undergone five courses of UFT and leucovorin as adjuvant chemotherapy. In May 2018, a laparoscopic hepatic left lateral segmentectomy was performed to treat a liver tumor, and a pathological diagnosis of colorectal liver metastasis was made. In August 2018, contrast-enhanced computed tomography (CT) revealed multiple lung metastases (Fig. 1a–c). The patient was referred to our hospital for systemic chemotherapy in his hometown.

**Fig. 2:**
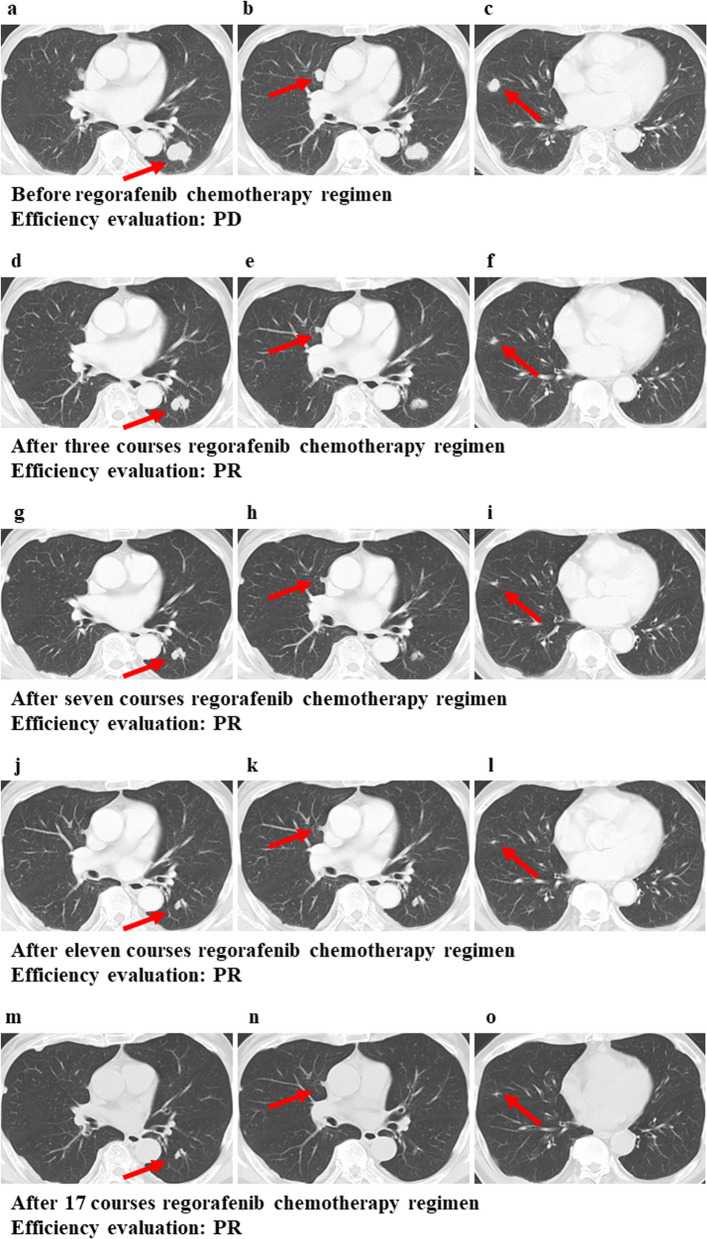
Consecutive CT findings from the start of third-line chemotherapy to the end of 17 courses. **a**–**c** The lung tumors had progressed after 11 courses of the FOLFIRI + RAM chemotherapy regimen had been administered. **d**–**f** The lung tumors had shrunk after three courses of the regorafenib chemotherapy regimen. **g**–**i** The lung tumors had shrunk after seven courses of the regorafenib chemotherapy regimen. **j**–**l** The lung tumors had shrunk after eleven courses of the regorafenib chemotherapy regimen. **m**–**o** The lung tumors had shrunk after 17 courses of the regorafenib chemotherapy regimen. These arrows indicate lung metastases

We started chemotherapy based on the Japanese Society for Cancer of the Colon and Rectum’s guidelines 2019 [8]. The patient had undergone 18 courses of the FOLFOX + bevacizumab (BV) chemotherapy regimen as their first-line therapy. After these cycles had been administered, CT revealed that the lung tumors had shrunk (Fig. 1d–f), and this was considered a partial response (PR) based on the Response Evaluation Criteria in Solid Tumors (RECIST) guidelines Ver. 1.1 [9]; however, this regimen was discontinued due to anaphylactic reactions as a side-effect of oxaliplatin. The patient was administered 11 courses of the FOLFIRI + ramucirumab (RAM) chemotherapy regimen as their second-line therapy, although the fourth and fifth courses were administered as part of the FOLFIRI chemotherapy regimen because of proteinuria. After 11 courses of the FOLFIRI + RAM chemotherapy regimen had been administered, CT revealed that the lung tumors had progressed (Fig. 2a–c), this was deemed a progressive disease based on the RECIST guidelines.

**Fig. 3:**
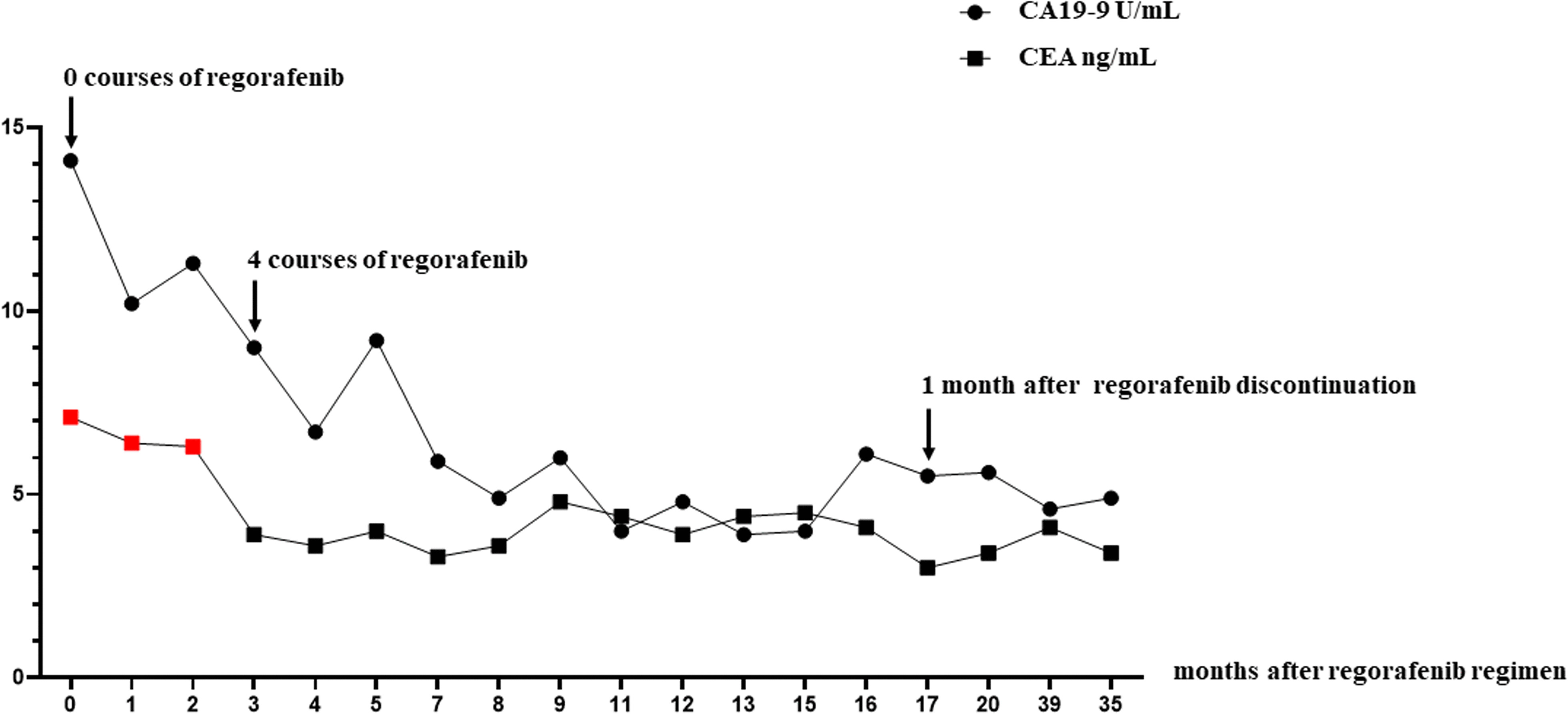
Changes in tumor markers (CEA and CA19-9). Red dots indicate values outside the normal range

**Fig. 4:**
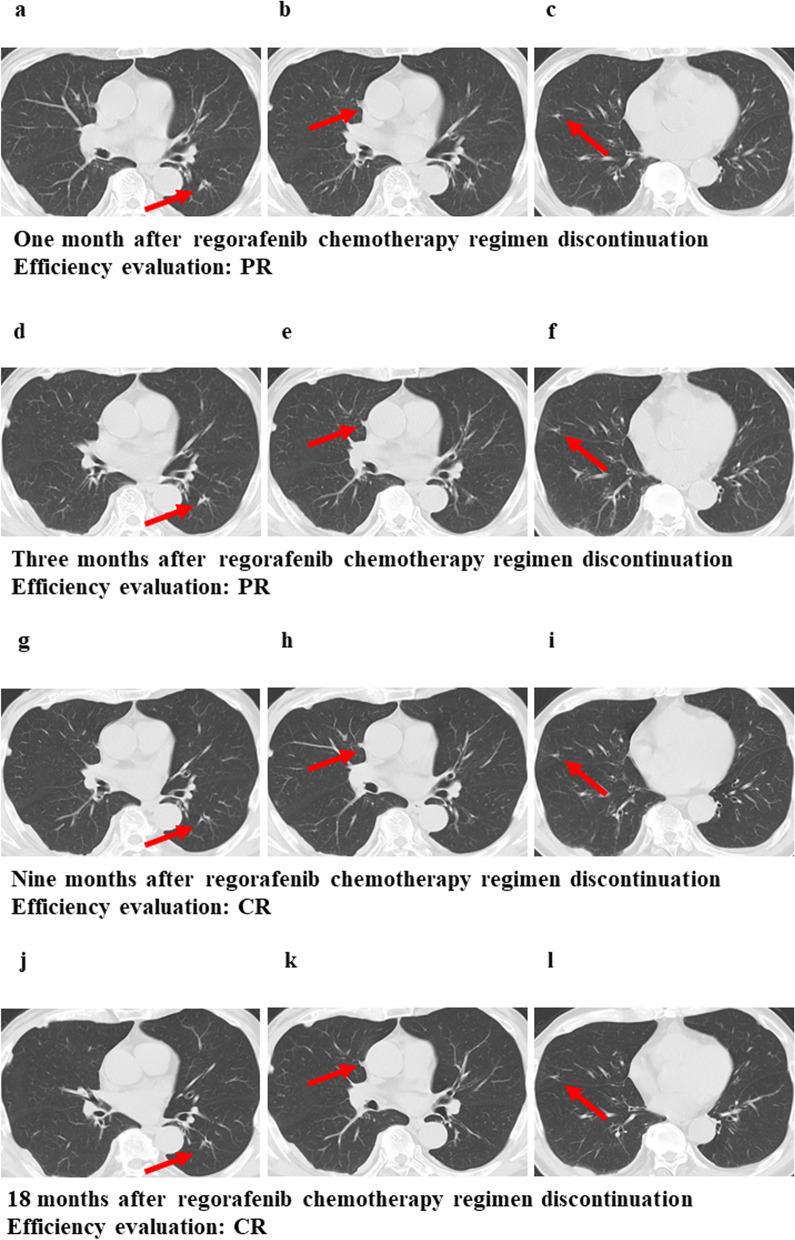
Consecutive CT findings after chemotherapy discontinuation. **a**–**c** The lung tumors had shrunk 1 month after chemotherapy discontinuation. **d**–**f** The lung tumors had shrunk 3 months after chemotherapy discontinuation. **g**–**i** The lung tumors had scarred 9 months after chemotherapy discontinuation. **j**–**l** The lung tumors had scarred 18 months after chemotherapy discontinuation. These arrows indicate lung metastases

The patient was administered the regorafenib chemotherapy regimen as their third-line therapy, starting with 120 mg per day for 3 weeks and 1 week of rest for one course, which was quickly reduced to 120 mg per day for 2 weeks and 1 week of rest after two courses based on the Common Terminology Criteria for Adverse Events (CTCAE version 5), indicating a grade 2 platelet count decrease [10] and bleeding of the gums. We evaluated the effects of the chemotherapy treatment effect on the lung tumors using CT after 3 (Fig. 2d–f), 7 (Fig. 2g–i), 11 (Fig. 2j–l), and 17 courses of the regorafenib chemotherapy regimen (Fig. 2m–o), and the lung tumors were found to have shrunk with time. Thus, they were each considered a PR according to the RECIST guidelines. The carcinoembryonic antigen (CEA) decreased during the regorafenib treatment and entered the normal range. Although the carbohydrate antigen 19-9 (CA19-9) was normal from the beginning of the treatment, it decreased during the regorafenib treatment (Fig. 3). After 19 courses of the regorafenib chemotherapy regimen, the estimated glomerular filtration rate (eGFR) declined below 40 mL/min/1.73 m^2^; thus, regorafenib was suspended until recovery, and the dose was reduced from 120 to 80 mg. After 21 courses of the regorafenib chemotherapy regimen, the chemotherapy was discontinued in response to the patient’s wishes. Even at 1 and 3 months after the discontinuation of the chemotherapy, CT revealed that the lung tumors had shrunk (Fig. 4a–f), with each considered a PR according to the RECIST guidelines. Furthermore, 9 months after the discontinuation of the chemotherapy, CT revealed scarring of the lung tumors (Fig. 4g–i). This was considered a CR, rather than PR, according to the RECIST guidelines. The patient remains disease-free 18 months after the discontinuation of chemotherapy. ^18^F-fluorodeoxyglucose positron emission tomography (PET)-CT was not performed because the patient’s consent could not be obtained.

## Discussion

To the best of our knowledge, this is the second case in which radiological CR has been identified in a patient with metastatic CRC who was treated with regorafenib. Few publications have described the detailed clinical course of patients with metastatic CRC that is respondent to regorafenib [7, 11–13], although some reports have addressed prognostic factors related to outcomes with regorafenib. In the REBACCA trial, survival was independently and unfavorably affected by the following variables: poor performance status, short time from initial diagnosis of metastases to the start of regorafenib, low initial regorafenib dose, > 3 metastatic sites, presence of liver metastases, and KRAS mutations. The study identified prognostic groups of patients with a low, intermediate, and high risk of death with a median survival of 9.2, 5.2, and 2.5 months, respectively [14]. According to the REBACCA trial, our case was considered to be in a prognostic group of patients with an intermediate risk of death. Sang et al. reported that PFS with regorafenib was significantly more prevalent in the left-sided CRC group than in the right-sided CRC group in a subpopulation with wild-type KRAS [15]. Regorafenib may have improved treatment outcomes based on appropriate biomarker studies.

In our case, rather than discontinuing chemotherapy after identifying it as CR, it was determined to be CR after the discontinuation of the chemotherapy. It was highly difficult to determine when to discontinue chemotherapy. The first case of metastatic CRC that showed a CR to regorafenib was considered a CR based on CT, PET-CT, and a tumor marker [7]. If a lack of recurrence had been indicated by PET-CT in our case, it would have been more suggestive of a CR, and a CR may have been determined earlier. In cases such as ours, the only rationale for discontinuing chemotherapy may be the confirmation of the disappearance of fluorodeoxyglucose uptake via PET-CT or the determination of pathological CR through surgery. However, seven lesions of colorectal cancer liver metastases displayed a radiological CR to the neoadjuvant chemotherapy on PET-CT; of these, 6 still contained viable tumor based on a histopathologic examination [16]. Thus, through follow-up should perhaps be performed, even in patients who have undergone PET-CT and obtained a radiological CR. Although there have been some reports of long-term survival among cases in which pulmonary resection is possible after chemotherapy, the resection rate is exceptionally low compared to that for liver metastases [17, 18]. Therefore, there is still no consensus regarding surgery for cases in which pulmonary resection becomes possible after chemotherapy.

## Conclusion

In this paper, we presented a second case of a radiological CR with regorafenib for multiple lung metastases of ascending colon cancer. The number of such cases is expected to increase with advances in chemotherapy. Further research is required to demonstrate the effectiveness of surgery and to determine for what length of time chemotherapy should be given to patients with radiological CR.

## Data Availability

Not applicable.
